# Eribulin mesylate exerts specific gene expression changes in pericytes and shortens pericyte-driven capillary network *in vitro*

**DOI:** 10.1186/2045-824X-6-3

**Published:** 2014-03-01

**Authors:** Sergei I Agoulnik, Satoshi Kawano, Noel Taylor, Judith Oestreicher, Junji Matsui, Jesse Chow, Yoshiya Oda, Yasuhiro Funahashi

**Affiliations:** 1 Eisai Inc, 4 Corporate Drive, Andover, MA 01810, USA; 2 Eisai Co., Ltd, Tokodai 5-1-3, Tsukuba, Ibaraki 300-2635, Japan; 3 Present address: Infinity Pharmaceuticals, 780 Memorial Drive, Cambridge, MA 02139, USA

**Keywords:** Eribulin, Endothelial cells, Pericytes, Gene expression profiling, Co-culture assay, Angiogenesis

## Abstract

**Background:**

Eribulin mesylate is a synthetic macrocyclic ketone analog of the marine sponge natural product halichondrin B. Eribulin is a tubulin-binding drug and approved in many countries worldwide for treatment of certain patients with advanced breast cancer. Here we investigated antiproliferative and antiangiogenic effects of eribulin on vascular cells, human umbilical vein endothelial cells (HUVECs) and human brain vascular pericytes (HBVPs), *in vitro* in comparison with another tubulin-binding drug, paclitaxel.

**Methods:**

HUVECs and HBVPs were treated with either eribulin or paclitaxel and their antiproliferative effects were evaluated. Global gene expression profiling changes caused by drug treatments were studied using Affymetrix microarray platform and custom TaqMan Low Density Cards. To examine effects of the drugs on pericyte-driven *in vitro* angiogenesis, we compared lengths of capillary networks in co-cultures of HUVECs with HBVPs.

**Results:**

Both eribulin and paclitaxel showed potent activities in *in vitro* proliferation of HUVECs and HBVPs, with the half-maximal inhibitory concentrations (IC_50_) in low- to sub-nmol/L concentrations. When gene expression changes were assessed in HUVECs, the majority of affected genes overlapped for both treatments (59%), while in HBVPs, altered gene signatures were drug-dependent and the overlap was limited to just 12%. In HBVPs, eribulin selectively affected 11 pathways (p < 0.01) such as Cell Cycle Control of Chromosomal Replication. In contrast, paclitaxel was tended to regulate 27 pathways such as PI3K/AKT. Only 5 pathways were commonly affected by both treatments. In *in vitro* pericyte-driven angiogenesis model, paclitaxel showed limited activity while eribulin shortened the formed capillary networks of HUVECs driven by HBVPs at low nmol/L concentrations starting at day 3 after treatments.

**Conclusions:**

Our findings suggest that pericytes, but not endothelial cells, responded differently, to two mechanistically-distinct tubulin-binding drugs, eribulin and paclitaxel. While eribulin and paclitaxel induced similar changes in gene expression in endothelial cells, in pericytes their altered gene expression was unique and drug-specific. In the functional endothelial-pericyte co-culture assay, eribulin, but not paclitaxel showed strong efficacy not only as a cytotoxic drug but also as a potent antivascular agent that affected pericyte-driven *in vitro* angiogenesis.

## Background

Formation and maintenance of new vascular vessels supporting tumor growth is a process which involves complex communications among different cell types. Interactions between endothelial cells and supporting pericytes and vascular smooth muscle cells lead to active remodeling during angiogenesis [1,2]. It is now well established that pericytes participate in angiogenic signal transduction and direct control of endothelial cell proliferation and thus play an important role in vascular morphogenesis and function [3]. Paracrine pericyte-endothelial interactions through VEGF, PDGF, and angiopoitin signaling promote tumor angiogenesis, pericyte recruitment and pericyte coverage of tumor vessels [4-7]. It is also known that pericytes shield endothelial cells from apoptotic signals [8]. As such, pericytes, in conjunction with endothelial cells, present a logical target for antiangiogenic therapeutic strategies.

Several approaches are currently employed to target tumor angiogenesis. Inhibition of VEGF signaling by specific antibodies or small molecule agents directed against VEGFR2 tyrosine kinase has been shown to inhibit formation and outgrowth of new blood vessels and several angiogenesis inhibitors have been used as anticancer therapy in patients [9]. In addition to inhibition of angiogenesis, recent studies showed that pharmacological VEGF inhibition, especially with anti-VEGF antibody, normalizes vessel structure and function through pericyte recruitment to disorganized tumor vessels [10,11]. Since normalization of tumor vasculature improves their perfusion and oxygenation, cytotoxic drugs were given in combination with anti-VEGF antibody causing the best response to radiation or cytotoxic therapy [12]. On the other hand, roles of pericytes in resistance to antiangiogenesis therapy have emerged, since most of cancer patients acquire resistance and become refractory to anti-VEGF therapy [13].

Another approach which is used to selectively target abnormal tumor vasculature is to apply agents which show vascular-disrupting activity to already formed blood vessels. Tumor vascular disrupting agents (VDA) cause selective induction of apoptosis in endothelial cells and induce vascular damage in already formed tumors [14]. Several specific VDAs are now undergoing clinical evaluation [15], although no VDA has been approved for cancer therapy.

Tubulin-binding agents were known to show antivascular effects represent both antiangiogenesis and vascular disrupting activity, which were well reported for paclitaxel and combrestatin analogs, respectively [16,17]. It is apparent that microtubules play an important role in angiogenesis and maintenance and stability of tumor vessels. Eribulin mesylate (eribulin), a non-taxane inhibitor of microtubule dynamics, belongs to the halichondrin class of antineoplastic drugs [18]. Eribulin’s novel mode of inhibiting microtubule dynamics differs from most other tubulin-targeting agents, and involves inhibition of the microtubule growth phase without affecting the shortening phase, together with sequestration of tubulin into non-productive aggregates [19]. This results in blockage of normal mitotic spindle formation, irreversible mitotic block and cell death by apoptosis [18,20,21]. At the biochemical level, eribulin achieves these results by specifically binding with high affinity to a small number of sites on the plus ends of microtubules [22].

In this paper we examined effects of eribulin on HUVECs and HBVPs *in vitro*. We analyzed effects of eribulin on global gene expression in HUVECs and HBVPs in a comparison with paclitaxel, which is a stabilizer of microtubule dynamics and has a distinct mechanism from eribulin. We determined effects of these two drugs on cell proliferation in mono-cultures of HUVECs and HBVPs, and employed a newly developed capillary network formation assay, in which HBVPs co-cultured with HUVECs to promote network formation, in order to assess antivascular activities of both drugs in the context of physiologically relevant cell-cell interactions.

## Materials and methods

### Cell cultures and compounds

Primary human umbilical vein endothelial cells (HUVECs) were either purchased from Lonza (Walkersville, MD) or isolated from a single umbilical cord by a method described previously [23] and maintained in endothelial cell growth medium EGM-2 supplemented with EGM-2 SingleQuots except for hydrocortisone (Lonza,). Human brain vascular pericytes (HBVPs) were obtained from ScienCell Research Laboratories (Carlsbad, CA), and were grown in Pericyte Medium (ScienCell). To confirm their authenticity, cultured HBVPs were examined for expression levels of 6 key pericyte markers grown on plastic (Additional file 1). Both HUVECs and HBVPs were grown on collagen type I-coated plastic ware. For cell proliferation and gene expression experiments, cells were used at <5 passages.

Green fluorescent protein (AcGFP)-expressing HUVECs were established by infection with a retrovirus for gene transfer of AcGFP followed by collecting high level AcGFP-expressing HUVECs by fluorescence activated cell sorting. Cells were maintained at 37°C in a humidified atmosphere containing 5% CO_2_.

Paclitaxel was purchased from Sigma-Aldrich (Saint Louis, MO) and Wako Pure Chemical (Osaka, Japan). Eribulin mesylate was manufactured by Eisai Co., Ltd (Ibaraki, Japan). Both compounds were dissolved in DMSO to yield a stock concentration of 1 mmol/L.

### Cell proliferation assay

HUVECs and HBVPs were plated at 3000 cells per well in 96-well plates. Three hours later serial dilutions of tested compounds were added. Control wells were treated with 0.1% DMSO. Cell growth was assessed 4 days later using the CellTiter-Glo Luminescent Cell Viability Assay (Promega, Madison, WI). Three experiments were performed, each in triplicate. The mean of the half maximal inhibitory concentration (IC_50_) value and 95% confidence interval (CI) were calculated based on IC_50_ values generated from separate sigmoidal curves representing the growth inhibition activity versus the eribulin and paclitaxel concentration of three independent experiments. Statistical analyses were performed using the GraphPad Prism version 5.02 (GraphPad Software, San Diego, CA).

### HUVEC/HBVP co-culture assay and measurement of pericyte-covered capillary network length reduction

HBVPs and AcGFP-expressing HUVECs were diluted and mixed to densities of 1.87 × 10^5^ cells/mL and 1.3 × 10^4^ cells/mL with medium, respectively. Cell suspensions were dispensed at 100 μL per well in black-walled, clear-bottomed, collagen type I-treated 96-well plates (Greiner Bio One, Frickenhausen, Germany) and incubated for 10 days with culture medium changes every 2 days.

To measure effects of compounds on capillary network lengths in HUVEC/HBVP co-cultures, 100 μL solutions of eribulin, paclitaxel or 0.1% DMSO (vehicle control) were exchanged into each well and incubated for 5 days. Three independent experiments were performed in triplicate. Fluorescence microscopy was performed every day for 5 days using an IN Cell Analyzer 1000 (GE Healthcare, Piscataway, NJ) to obtain images of pericyte-covered capillary networks formed by AcGFP-expressing HUVECs under co-culture conditions. Images were negative/positive inverted and high-contrasted by Irfan View software version 3.61 (Irfan Skiljan, Wierner Neustadt, Austria), followed by analysis using Angiogenesis Image Analyzer software version 2.0 (Kurabo, Osaka, Japan) to measure lengths of pericyte-covered capillary networks. Data for pericyte-covered capillary network lengths were expressed as means + SEM.

IC_50_’s of 5-day treatments in three independent experiments were analyzed by nonlinear regression analysis, and means of three IC_50_ values were determined. Statistical analyses were performed using GraphPad Prism version 5.04 (GraphPad Software).

### Measurement of cell viability in co-culture

Assessment of cell viability in the co-culture assay was performed by modification of the 3-(4,5-dimethylthiazol-2-yl)-2,5-diphenyltetrazolium bromide colorimetric assay [24]. After measurement of pericyte-covered capillary network lengths in the HUVEC/HBVP co-culture assay, medium in wells was exchanged with 100 μL of 11-fold media-diluted WST-8 reagent (Cell Counting Kit-8, Dojindo Laboratories, Kumamoto, Japan), followed by incubation for one additional hour. The optical density (OD) at 450 nm of each well was measured with a microplate reader (SpectraMax 250, Molecular Devices, Sunnyvale, CA), with a reference wavelength of 650 nm. Data for cell viability were expressed as means + SEM.

### Microarray analysis

HUVECs and HBVPs were plated at 1 × 10^5^ cells per well in 12-well plates. The next day, cells were treated with compounds at 10 × IC_50_ concentrations as determined in cell proliferation assays. Control wells were treated with 0.1% DMSO in culture media. Experiments were done in triplicates. Twenty four hours later, cells were collected and RNA was extracted using an RNeasy Mini kit (Qiagen, Valencia, CA) with DNase I on-column treatment according to the manufacturer’s protocol. RNA was quantified using Nanodrop ND-1000 (Thermo Scientific, Wilmington, DE). For each sample, 1 μg total RNA was used to prepare biotinylated fragmented cDNA for analysis on Human Exon 1.0 ST arrays (Affymetrix, Santa Clara, CA). Sample preparation was performed in accordance with manufacturer’s instructions using the WT Expression kit (Life Technologies, Carlsbad, CA) and the GeneChip WT (Whole Transcript) Terminal Labeling Kit (Affymetrix). Hybridizations were conducted according to the GeneChip Expression Analysis Technical Manual (Affymetrix). Arrays were washed and stained using Affymetrix Fluidics Station 450, and finally scanned using Affymetrix GeneChip Scanner 3000.

Gene chips were analyzed using Affymetrix Microarray Analysis Suite (MAS) version 5.0 to obtain raw data. Fluorescence intensities of scanned images were quantified, corrected for background noise, and RMA normalized using Refiner software (Genedata, Basel, Switzerland). Normalization procedure within RMA consisted of a GC background subtraction, quantile normalization and summarization. QC analysis of the microarrays was performed according to the standard protocol within Genedata software. All microarrays passed QC criteria. Statistical analysis was performed with Expressionist software (Genedata). We restricted our analysis to gene intensities >100, based on the detection limit of the Affymetrix gene chip. Gene expression levels of untreated samples were compared to those from treated samples using *t*-test analysis. Comparisons were limited to p-values of 0.05 in order to eliminate 95% of false positives from the data set, and to fold change levels of at least 1.5 in order to remove background effects.

### Quantitative real time PCR analysis

Reverse transcription was carried out with 2.5 μg of total RNA using a Superscript VILO kit (Life Technologies). Synthesized cDNA were used as templates for quantitative polymerase chain reaction (qPCR) using custom TaqMan Low Density Array (TLDA) (Life Technologies) with ABI 7900HT (Life Technologies). Selected gene probes related to differentially expressed genes identified in the microarray experiment were used for TLDA (Additional file 2). Data were normalized using Expressionist (Genedata).

Hierarchical clustering was done using Genedata software. Genes differentially expressed between eribulin and control and between paclitaxel and control were uploaded into Ingenuity Pathway Analysis (IPA; Ingenuity Systems, Redwood City, CA), and only significantly affected signaling pathways were reported with the cut off value of p < 0.01 for pathway enrichment.

## Results

### Effects of eribulin and paclitaxel on cell proliferation *in vitro*

Tubulin-binding drugs exert their effects on actively dividing cells by disrupting normal mitotic spindle formation and thus causing cell cycle blockage in mitosis leading ultimately to cell death. It was previously shown that eribulin is a highly active compound in growth inhibition of numerous cancer cells [18]. To evaluate drug effects on proliferation of HUVECs and HBVPs, we tested eribulin and paclitaxel in a standard cell proliferation assay. Table 1 shows the results of this analysis. Compared to HBVPs, both compounds were more active against HUVECs, showing similar IC_50_ values in the sub-nmol/L range: 0.54 for eribulin and 0.41 for paclitaxel. HBVPs were more resistant to both eribulin and paclitaxel, with IC_50_ values of 1.19 and 2.19 nmol/L, respectively.

**Table 1:** Inhibition of HUVEC and HBVP cell growth by eribulin and paclitaxel

	**Growth inhibition, IC**_**50 **_**(nmol/L)**	
**Cell type**	**Eribulin**	**Paclitaxel**
HUVEC	0.54 ± 0.09	0.41 ± 0.12
HBVP	1.19 ± 0.20	2.19 ± 0.55

### Gene expression analysis of eribulin- and paclitaxel-treated HUVECs and HBVPs

To evaluate transcriptional changes in HUVECs and HBVPs caused by treatment with eribulin and paclitaxel, we used whole human genome microarrays. We selected comparable functional concentrations of both compounds based on their activities in cell growth inhibition assays (10 × IC_50_) for 24 hours treatment based on the number of genes altered after comparing several different conditions such as concentration and duration of treatments.

**Figure 1:**
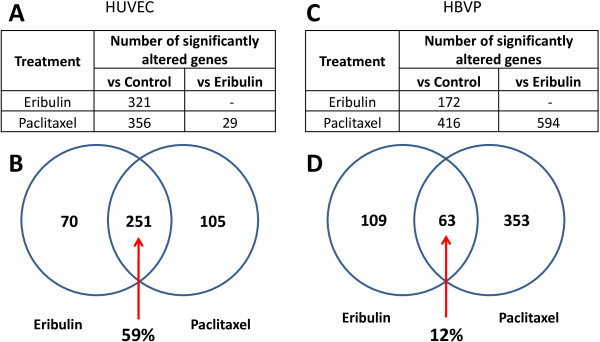
**Gene expression analysis of vascular cells treated with eribulin and paclitaxel. A**. Number of differentially expressed genes in HUVECs treated with eribulin or paclitaxel. **B**. Comparison of eribulin and paclitaxel gene signatures in HUVECs by Venn diagram. **C**. Number of differentially expressed genes in HBVPs. **D**. Comparison of eribulin and paclitaxel gene signatures in HBVPs by Venn diagram. Analysis was restricted to genes with signal intensities >100, p-values < 0.05 and fold change levels of at least 1.5 in order to remove background effects.

In HUVECs, eribulin significantly altered expression of 321 genes compare to control vehicle-treated cells (Figure 1A). Paclitaxel caused transcriptional changes of 356 genes compare to control. When we compared these two signatures, more than 59% of genes overlapped between the two groups (Venn diagram, Figure 1B). It should be noted that all 251 overlapping genes changed expression in the same direction in eribulin and paclitaxel treated cells: 235 genes were down-regulated while 16 were up-regulated after both treatments. Interestingly, direct comparison of eribulin versus paclitaxel treatments showed significantly different expression changes in only 29 genes. Both treatments led to down-regulation, not up-regulation, of a majority of the affected genes in HUVECs (Additional file 3).

A different pattern of expression changes was observed in HBVPs with both drug treatments. Eribulin altered expression levels of 172 genes while paclitaxel changed expression of 416 genes compared to vehicle-treated cells (Figure 1C). Only 63 genes (12%) of the total number of 525 altered genes overlapped between the two drugs (Figure 1D). Eribulin caused down-regulation of 105 genes and up-regulation of 67 genes, while paclitaxel led to higher number of up-regulated genes (348) compared to down-regulated genes (68) (Additional file 3). Direct comparison of eribulin- and paclitaxel-treated changes of gene expression profiling showed a significant number of genes (594) differentially altered in HBVPs. These data indicate that, in contrast to HUVECs, transcriptional changes in HBVP were very drug-specific.

Comparison of gene profiles in HUVECs and HBVPs revealed only 14 altered genes that were common to all treatments. Four of these, BTG2, CDC45, CSE1 and MCM5, were cell cycle related genes. The largest group of 6 genes among the 14 common genes consisted of 5 linker histone H1 gene family members (HIST1H1A, HIST1H1B, HIST1H1D, HIST1H2AB/HIST1H2AE and HIST1H2BM) and one nucleosome histone H2 gene (HIST2H2AC). In addition, anti-silencing function 1B histone chaperone (ASF1B) was also among commonly effected genes.

To evaluate differential effects of eribulin and paclitaxel on pericytes in more details, we performed pathway analysis using Ingenuity software (Ingenuity Pathway Analysis) (Table 2). Results showed that eribulin significantly affected pathways related to cell cycle control of chromosomal replication, RAN signaling, as well as polyamine regulation and mismatch repair. On the other hand, paclitaxel regulated PI3K/AKT, HGF and WNT pathways as well as stress response and p53 signaling pathways and others. These results indicate that pericyte functions in general might be affected differently by eribulin and paclitaxel.

**Table 2:** Signaling pathways affected by eribulin and paclitaxel in HBVPs

**Eribulin affected pathways**	**p values (-log)**	**Paclitaxel affected pathways**	**p values (-log)**
Cell cycle control of chromosomal replication	6.85E+00	PI3K/AKT signaling	5.91E+00
Granzyme A signaling*	6.27E+00	Hepatic fibrosis/hepatic stellate cell activation*	4.59E+00
RAN signaling	3.37E+00	NRF2-mediated oxidative stress response	4.11E+00
Polyamine regulation in colon cancer	3.03E+00	Glucocorticoid receptor signaling	4.07E+00
Role of macrophages, fibroblasts and endothelial cells in rheumatoid arthritis*	2.79E+00	Aryl hydrocarbon receptor signaling	3.89E+00
Airway pathology in chronic obstructive pulmonary disease	2.68E+00	IGF-1 signaling	3.82E+00
IL-17A signaling in fibroblasts*	2.44E+00	p53 signaling	3.79E+00
Hepatic fibrosis/hepatic stellate cell activation*	2.10E+00	Granzyme A signaling*	3.30E+00
Bladder cancer signaling*	2.09E+00	Cell cycle regulation by BTG family proteins	3.19E+00
Mismatch repair in eukaryotes	2.06E+00	Aldosterone signaling in epithelial cells	2.95E+00
Putrescine degradation III	2.01E+00	MIF regulation of innate immunity	2.87E+00
		CDK5 signaling	2.70E+00
		Wnt/b-catenin signaling	2.60E+00
		Role of macrophages, fibroblasts and endothelial cells in Rrheumatoid arthritis*	2.42E+00
		HGF signaling	2.38E+00
		MIF-mediated glucocorticoid regulation	2.38E+00
		Estrogen receptor signaling	2.37E+00
		Role of IL-17A in arthritis	2.34E+00
		VDR/RXR activation	2.31E+00
		Role of CHK proteins in cell cycle checkpoint control	2.30E+00
		IL-17A signaling in fibroblasts*	2.28E+00
		Stearate biosynthesis I (animals)	2.28E+00
		Superoxide radicals degradation	2.24E+00
		Apoptosis signaling	2.06E+00
		CD40 signaling	2.06E+00
		Bladder cancer signaling*	2.03E+00
		Melanoma signaling	2.00E+00

Genes with significantly changed expression levels compare to controls were analyzed using Ingenuity Pathway Analysis. Cell signaling pathways significantly affected by eribulin or paclitaxel treatments are shown.

*Common pathways for both treatments.

**Figure 2:**
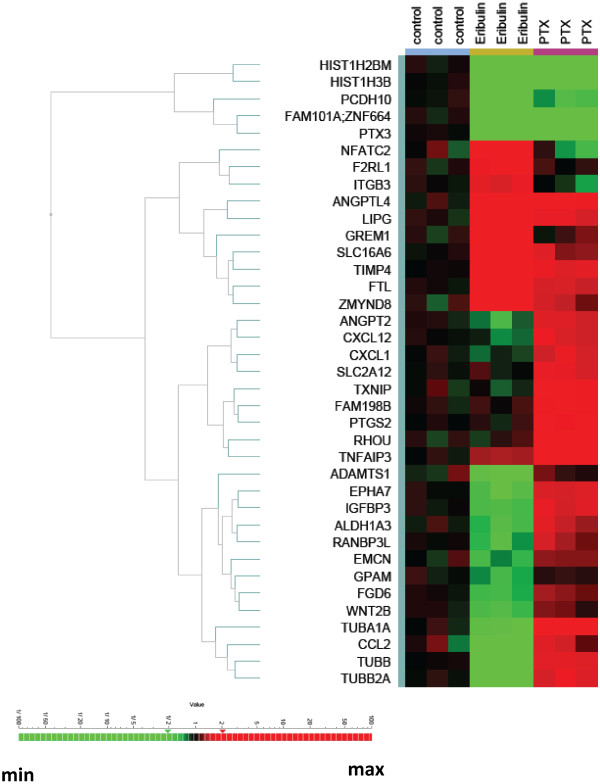
**Hierarchical clustering of differentially expressed genes in HBVPs.** HBVPs were treated with eribulin or paclitaxel (PTX) for 24 hours in triplicates. Expression levels of selected genes identified as differentially expressed in microarray experiment were analyzed by qPCR using custom TLDAs. Genes were clustered according to their expression patterns.

We confirmed a subset of genes differentially expressed in HBVPs after treatment with eribulin versus paclitaxel by qPCR using custom TLDA (Figure 2, Additional file 2). We showed that expression of 37 genes was significantly changed in both of eribulin and paclitaxel treatments as measured by qPCR. Furthermore, expression alterations of several genes were confirmed by Western blot analysis (Additional file 4). These altered genes formed drug-specific signatures and were clustered according to the treatment. Three major clusters of genes were identified, one with downregulated genes for both eribulin and paclitaxel (5 genes) and another one with upregulated genes for both treatments (10 genes), but altered levels with eribulin treatment were more robust. The third group with downregulated genes for eribulin and upregulated genes for paclitaxel signature contained largest numbers of genes (22 genes) clustered into two groups, one with ANGPT2, CXCL12, CXCL1, SLC2A12, TXNIP, FAM198B, PTGS2, RHOU, TNFAIP3 genes and another with ADMTS1, EPHA7, IGFBP3, ALDH1A3, RANBP3L, EMCN, GPAM, FGD6, WNT2B, TUBA1A, CCL2, TUBB, TUBB2A genes.

### Effects of eribulin and paclitaxel on capillary networks in endothelial cell-pericyte co-cultures

**Figure 3:**
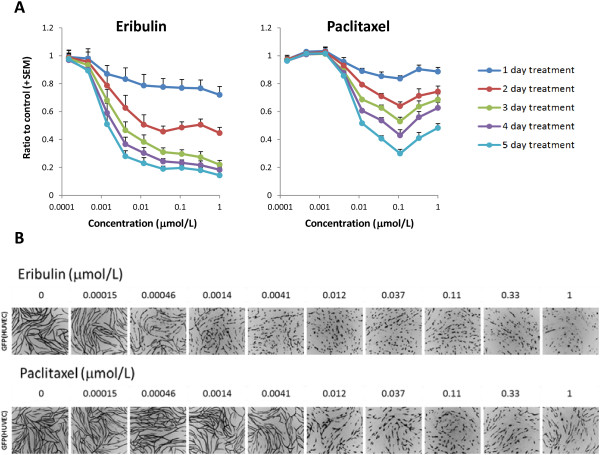
**Pericyte-covered capillary network length alterations by eribulin and paclitaxel in HUVEC and HBVP co-culture assay. A**. HUVECs and HBVPs in co-culture were treated with eribulin or paclitaxel for 5 days and pericyte-covered capillary network lengths were calculated by the length of networks formed by HUVECs. Data represent means + SEM from three independent experiments. **B**. Representative images of pericyte-covered capillary network captured on 5-day treatment. AcGFP-expressing HUVECs form networks (in black) which are responding to the drug treatments by shortening of their lengths.

We examined effects of eribulin and paclitaxel on pericyte-covered capillary networks in a unique assay system in which HUVECs and HBVPs were co-cultured. In this assay system, AcGFP-expressing HUVECs form pericyte-covered capillary networks driven by HBVPs, since HUVECs mono-culture in the 2D condition never formed networks without pericytes or fibroblasts (data not shown), and the length of these networks can be measured and analyzed after drug treatment. Figure 3 shows pericyte-covered capillary network length alterations by the treatment with eribulin or paclitaxel. In both groups, pericyte-covered capillary network lengths were decreased in a time-dependent manner and maximum at 5 days after treatment. IC_50_ values at 3- and 5-day treatments with eribulin were 3.6 nmol/L and 1.5 nmol/L, respectively, while with paclitaxel, IC_50_ values were >1 μmol/L at 3 days and 13 nmol/L at 5 days (Table 3). To evaluate effects of compounds on capillary networks formed with only HUVECs, we used standard sandwich tube formation assay (Additional file 5). Capillary network formation was induced by VEGF stimulation with HUVECs cultured between two layers of collagen gel without co-culturing with HBVPs. In this assay, eribulin and paclitaxel showed similar IC_50_ values (0.65 - 0.72 for eribulin and 1.18 - 1.26 for paclitaxel) in shortening capillary network at 4 days after treatment, which suggest that pericyte-covered condition is an important component of differential response of capillary network to the treatment with eribulin in co-culture assay compared to paclitaxel.

**Table 3:** Effects of eribulin and paclitaxel on capillary networks in HUVEC and HBVP co-cultures

**Treatment**	**Network shortening, IC**_**50 **_**(nmol/L)**				
	**Day 1**	**Day 2**	**Day 3**	**Day 4**	**Day 5**
Eribulin	>1,000	>1,000	3.6	2.1	1.5
Paclitaxel	>1,000	>1,000	>1,000	>1,000	13

**Figure 4:**
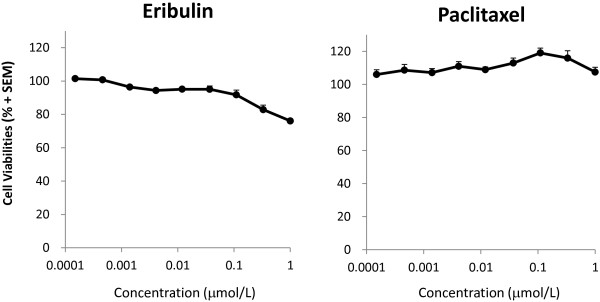
**Cell viabilities after treatment with eribulin or paclitaxel in HUVEC and HBVP co-cultures.** Cell viabilities were measured by the WST-8 assay in HUVEC/HBVP co-cultures treated with eribulin or paclitaxel for 5 days. Data represent means + SEM from three independent experiments.

Cell viability in the co-culture assay after 5 days of treatment with eribulin was slightly decreased (about 20%) (Figure 4). Since majority cells in co-culture were HBVPs, we assume that most of the signal in this assay came from pericyte cells. However, it might reflect shorter length of HUVECs network, because there was slightly higher cell viability in the co-culture assay compared to HBVPs mono-culture (Additional file 6). These data indicate that eribulin has greater antivascular effects on pericyte-covered capillary network shortening relative to its antiproliferative effects on HUVECs, whereas effects of paclitaxel on pericyte-covered capillary network shortening was much weaker than its antiproliferative effects on HUVECs.

## Discussion

In addition to normal development, angiogenesis plays an important role in tumor growth [25]. However, tumor vasculature is largely distinct from normal vessels in a number of properties. Tumor vasculature is known to be grossly disorganized and tortuous. Furthermore, tumor vasculature is leakier than normal vasculature because it grows fast and does not have close interactions with pericytes. Interactions between endothelial cells and pericytes in the blood vessel wall are important processes in the regulation of vascular formation, stabilization, remodeling and function. Failure of such interactions are implicated in human pathological conditions, including diabetic microangiopathy [26], ectopic tissue calcification [27], stroke [28] and cerebral autosomal dominant arteriopathy with subcortical infarcts and leukoencephalopathy (CADASIL) syndrome, one of the most common inherited small vessel diseases of the brain [29]. Tumor vasculature is heterogeneous in its interaction with pericytes, and many reports indicate that those having a “mature” appearance, defined primarily as having close endothelial cell-pericyte interactions, show resistance to antiangiogenic therapies [30].

To shed light on effects of eribulin on tumor angiogenesis, we studied HUVECs and HBVPs as models of endothelial cells and pericytes in tumor vasculature. We showed that these two cell types responded differently to eribulin and paclitaxel treatment by specific changes in expression of multiple genes. In HUVECs, both eribulin and paclitaxel led to down-regulation of most affected genes, with drug signatures greatly overlapping. Notably, eribulin and paclitaxel signatures in HBVPs were clearly different from those in HUVECs.

We found that among 14 genes common for both drugs and both cell types, 4 genes were related to cell cycle progression (BTG2, CDC45, CSE1 and MCM5). The largest group of 6 common genes, all of which were down-regulated, was made up of histones, with 5 of these belonging to the H1 gene family. In addition, 1B histone chaperone gene ASF1B was also down-regulated by both drugs in HUVECs and HBVPs. There are 11 known H1 family members in the human genome, and 5 of those were found in this study to be down-regulated by the two tubulin-binding drugs under study. The H1 histones work as linkers for nucleosomal core particles and have an important role in establishing and maintaining chromatin structure and regulation of gene activity [31]. Down-regulation of H1 histones causes de-condensation of chromatin and possibly up-regulation of gene transcription.

Our data showed that in HBVPs, gene expression changes caused by eribulin and paclitaxel were dramatically different. Only 12 percent of genes were similarly affected by both drugs, while the rest showing drug-specific changes. While paclitaxel induced transcriptional activity of a majority of affected genes, 61 percent of genes were down-regulated and by eribulin. Direct comparison of eribulin’s versus paclitaxel’s gene expression profiles showed a large number of differentially expressed genes. These data indicate that either the direction of altered expression was opposite for the two drugs, or the degrees of alteration were significantly different between eribulin and paclitaxel treatment.

A number of observed differentially expressed eribulin-specific genes (Additional file 3, highlighted in red) were previously reported to be associated with angiogenesis, pericyte biology and vascular remodeling (NOTCH3, PTX3,TNFAIP1, PGF, GREM1, TIMP4, LIPG, MYOCD) [32-36]. Of particular interest is NOTCH3, a gene encoding one of the notch family members and known to be underlying cause of CADASIL [37,38]. NOTCH signaling is critical for maintenance of normal vascular structure, angiogenesis and vascular remodeling in both physiological and pathological conditions [39,40]. In our study we detected significant upregulation of NOTCH3 expression after eribulin treatment in HBVPs.

To evaluate specific effects of eribulin on signaling pathways, we performed pathway analysis using Ingenuity software. This analysis showed that in pericytes, genes in several pathways were selectively affected by eribulin compared to paclitaxel. In particular, cell cycle control of chromosomal replication including RAN signaling and mismatch repair pathways were among most significantly changed.

Most studies published to date have used *in vitro* endothelial cell-based vascular disruption assays to evaluate activities of compounds against newly formed capillary-like structures. In such assays, endothelial cells that are plated on thick layers of Matrigel form networks of cord-like structures, reminiscent of newly formed vessels. Treatment with antivascular compounds results in disruption of the integrity of such cord-like networks. However, such assays, using only endothelial cells, do not take into account the physiologically-relevant close interactions between endothelial cells and supporting pericytes within the context of tumor vasculature. To overcome this limitation, we developed a novel assay in which AcGFP-transfected endothelial cells grow and form capillary networks in co-culture with pericytes. The effects of two tubulin-targeting compounds, eribulin and paclitaxel, on network formation were evaluated in this assay by measuring the lengths of pericyte-covered capillary networks. We found that eribulin and paclitaxel behaved dramatically different in this assay. Eribulin, in contrast to paclitaxel, showed significant antivascular activity causing dramatic pericyte-covered capillary network shortening effects relative to its antiproliferative effects on HUVECs. On the other hand, pericytes seemed to protect endothelial networks under paclitaxel treatment conditions and thus network shortening activity was much weaker compared to its antiproliferative effects on HUVECs. Thus, eribulin appeared to impair interaction of pericytes with endothelial cells through the activity against pericytes differently from paclitaxel in this assay. Consequently, eribulin showed much higher activity as an anti-vascular agent than to paclitaxel in this co-culture assay. Interestingly, in the sandwich tube formation assay in which HUVECs can form capillary network without co-culturing with HBVPs, eribulin and paclitaxel showed similar IC_50_ values in shortening capillary network at 4 days after treatment. This strongly supports the above hypothesis. In future studies, it will be important to compare and further define this newly discovered antipericyte-based antivascular property of eribulin with other known tubulin-binding drugs both in *in vitro* and *in vivo* angiogenesis models.

## Conclusions

Current study showed that eribulin, but not paclitaxel, causes dramatic shortening and interruption of pericyte-driven capillary networks formed by HUVECs in co-culture with HBVPs. The effect was observed at compound’s concentrations which did not induce significant toxicity in pericytes of confluent state. Gene expression profiling of pericytes treated with eribulin showed specific signature different from paclitaxel’s one. We propose that observed eribulin’s antiangiogenic effect in co-culture model is based on drug’s effects against pericytes.

## Competing interests

SIA, SK, NT, JM, JC, YO and YF are full-time employees of Eisai. JO was a full-time employee of Eisai. The authors declare no other competing interests.

## Authors’ contributions

All authors contributed to the design of the study, acquisition of data, analysis and interpretation of data or manuscript writing, and have read and approved the final manuscript.

## Supplementary Material

Additional file 1 **Expression of key pericyte markers in HBVPs and HUVECs.** Expression of α-SMA, Desmin (DES), CD248, NG2, CD146 and platelet derived growth factor receptor-beta (PDGFRB) genes (Chen et al., [34]) was analyzed in cultured HBVPs and HUVECs growing on plastic. All markers were highly expressed in HBVPs. At the same time, these genes were expressed at much lower level in HUVECs with the exception of CD146. Based on these data we conclude that HBVPs kept pericyte phenotype even growing on plastic. Click here for file

Additional file 2 **List of differentially expressed genes in HBVPs identified in the microarray experiment included in TLDA.** Seventy four selected genes from eribulin and paclitaxel pericyte signatures were analyzed by qPCR. Expression of housekeeping genes ACTB and GAPDH was used for normalization. In parenthesis are numbers of genes specific to eribulin, paclitaxel or both treatments versus control or each other. Click here for file

Additional file 3 **List of genes with significantly changed expression level in HUVECs and HBVPs after the treatment with eribulin or paclitaxel.** Genes with signal intensities >100, p-values < 0.05 and fold change levels of at least 1.5 compare to control are shown. Each treatment was conducted in triplicates. FC = Fold Change. Click here for file

Additional file 4 **Western blot analysis of selected proteins in HBVPs treated with eribulin or paclitaxel.** HBVPs were treated with eribulin or paclitaxel for 24 hours at 10 × IC_50_ concentrations as determined in cell proliferation assay. 0.1% DMSO was used as vehicle control. **A.** Expression levels of several proteins were analyzed by western blot analysis using the antibodies against α1A-tubulin (clone DM1A, Millipore, Billerica, MA), β-tubulin (Santa Cruz Biotechnology, Santa Cruz, CA), IGFBP3 (Santa Cruz Biotechnology) and β-actin (clone AC-15, Sigma Aldrich St. Louis, MO). **B.** The signals of protein bands were quantified using Multi Gauge version 3.0 software (Fuji Film, Tokyo, Japan). The quantitative data of α1A-tubulin, β-tubulin and insulin-like growth factor binding protein 3 were adjusted by the intensity of β-actin. The calculated values and the intensity of β-actin are also shown. Click here for file

Additional file 5 **Effect of eribulin and paclitaxel on angiogenesis in the sandwich tube formation assay.** An aliquot (0.4 mL) of the collagen gel mixture (Nitta Gelatin, Osaka, Japan) was added to each well of 24-well plates and allowed to gel at 37°C. HUVECs were harvested by trypsinization, counted and plated onto the gel at 1.5 × 10^5^ cells per well with human endothelial serum free medium (Life Technologies) containing 10 ng/mL of EGF and 20 ng/mL VEGF (assay medium). After overnight incubation at 37°C in a humidified atmosphere containing 5% CO_2_, medium was removed and 0.4 mL of collagen gel was added to the cells and incubated for 4 hr at 37°C. An aliquot (1.5 mL) of assay medium, containing 0.1% DMSO (as vehicle) or test compounds were added to each well. HUVECs sandwiched in collagen gel were incubated at 37°C in a humidified atmosphere containing 5% CO_2_ for 4 days and photomicrographs of capillaries were taken with a light microscope using BZ-9000 (Keyence, Osaka, Japan) after adding 0.4 mL of MTT solution. **A.** Tube length of each capillary was measured using Angiogenesis Image Analyzer software version 2.0 (Kurabo). Assays were performed in duplicate. Both compounds showed close IC_50_ values: 0.65 - 0.72 for eribulin and 1.18 - 1.26 for paclitaxel. **B.** Representative images are shown. Click here for file

Additional file 6 **OD**_**450 **_**values (cell viability) of HBVP mono-culture and HUVEC/HBVP co-culture.** In the HBVP mono-culture, HBVPs were diluted to densities of 1.87 × 10^5^ cells/mL. In the HUVEC/HBVP co-culture, HBVPs and AcGFP-expressing HUVECs were diluted and mixed to densities of 1.87 × 10^5^ cells/mL and 1.3 × 10^4^ cells/mL with medium, respectively. Cell suspensions were dispensed at 100 μL per well in 96-well plates and incubated for 15 days with culture medium changes every 2 days. **A.** Cell viabilities were measured by the WST-8 assay. HUVEC/HBVP co-culture showed slightly higher values compared to HBVP mono-culture, because of HUVEC viability. Data represent means + SEM from three independent experiments. **B.** Representative images are shown. Click here for file
