# Supplementary material for: Eribulin mesylate exerts specific gene expression changes in pericytes and shortens pericyte-driven capillary network in vitro
Source: Vasc Cell. 2014 Mar 1;6:3. doi: 10.1186/2045-824X-6-3 (PMC4016419; doi:10.1186/2045-824X-6-3)
Supplement: Additional file 1 — Expression of key pericyte markers in HBVPs and HUVECs. Expression of α-SMA, Desmin (DES), CD248, NG2, CD146 and platelet derived growth factor receptor-beta (PDGFRB) genes (Chen et al., [34]) was analyzed in cultured HBVPs and HUVECs growing on plastic. All markers were highly expressed in HBVPs. At the same time, these genes were expressed at much lower level in HUVECs with the exception of CD146. Based on these data we conclude that HBVPs kept pericyte phenotype even growing on plastic. [file 2045-824X-6-3-S1.pdf]

	mean Ct						
Cells	Endogenous Coltrol: ACTB	alpha-SMA	DES	CD248	NG2	CD146	PDGFRB
**HBVP**	16.92	24.58	25.90	20.72	19.56	25.31	21.07
**HUVEC**	17.7	26.88	35.23	30.90	36.99	20.07	33.01

	Relative expression to HBVP						
Cells	ACTB	alpha-SMA	DES	CD248	NG2	CD146	PDGFRB
**HBVP**	-	1.00	1.00	1.00	1.00	1.00	1.00
**HUVEC**	-	0.35	0.00	0.00	0.00	65.13	0.00
