# Supplementary material for: Eribulin mesylate exerts specific gene expression changes in pericytes and shortens pericyte-driven capillary network in vitro
Source: Vasc Cell. 2014 Mar 1;6:3. doi: 10.1186/2045-824X-6-3 (PMC4016419; doi:10.1186/2045-824X-6-3)
Supplement: Additional file 2 — List of differentially expressed genes in HBVPs identified in the microarray experiment included in TLDA. Seventy four selected genes from eribulin and paclitaxel pericyte signatures were analyzed by qPCR. Expression of housekeeping genes ACTB and GAPDH was used for normalization. In parenthesis are numbers of genes specific to eribulin, paclitaxel or both treatments versus control or each other. [file 2045-824X-6-3-S2.pdf]

Eribulin vs control (19)	Paclitaxel vs control (19)	Eribulin and Paclitaxel vs control (11)	Eribulin vs Paclitaxel (25)
ADAMTS1	ANGPT2	ANGPTL4	ALDH1A3
CCL2	C4orf18	FAM101A	ANAPC11
F2RL1	CAPN7	GCNT1	AP3S2
FTL	CXCL1	GDF15	BID
GPRC5B	EMCN	HIST1H2BM	CXCL12
GREM1	EPHA7	HIST1H3B	CYFIP1
H19	FAS	HIST1H3G	DAAM1
LIFR	FASN	HIST1H3H	FAU
LIPG	PECR	HIST1H4L	FGD6
NFATC2	PPP2R5A	PRDM15	FZD2
PCDH10	PTGS2	SERPINB2	GPAM
PGF	RANBP3L		IFNAR2
POLA2	RHOU		IGFBP3
PTX3	SLC2A12		IL1R1
SLC16A6	TMEM191A		ITGB3
SLC7A8	TNFAIP3		MAP3K5
TIMP4	TUBB		MSH2
TUBA1B	TUBB2A		PAIP1
ZMYND8	TXNIP		ROCK1
			RPL7L1
			ST7L
			TRIP11
			TUBA1A
			VEGFB
			WNT2B
