# Supplementary figures and images for: Eribulin mesylate exerts specific gene expression changes in pericytes and shortens pericyte-driven capillary network in vitro
Source: Vasc Cell. 2014 Mar 1;6:3. doi: 10.1186/2045-824X-6-3 (PMC4016419; doi:10.1186/2045-824X-6-3)

**A**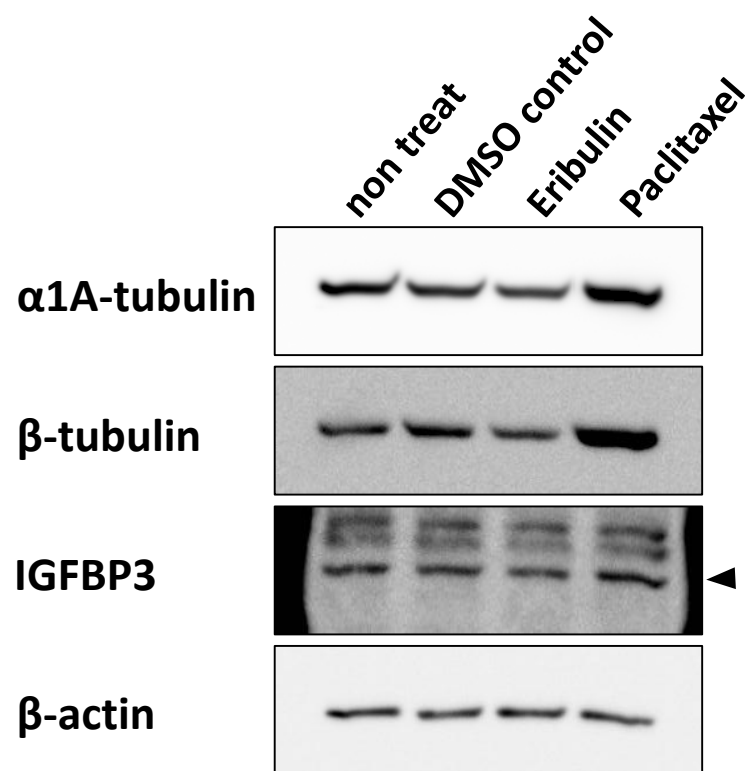**B**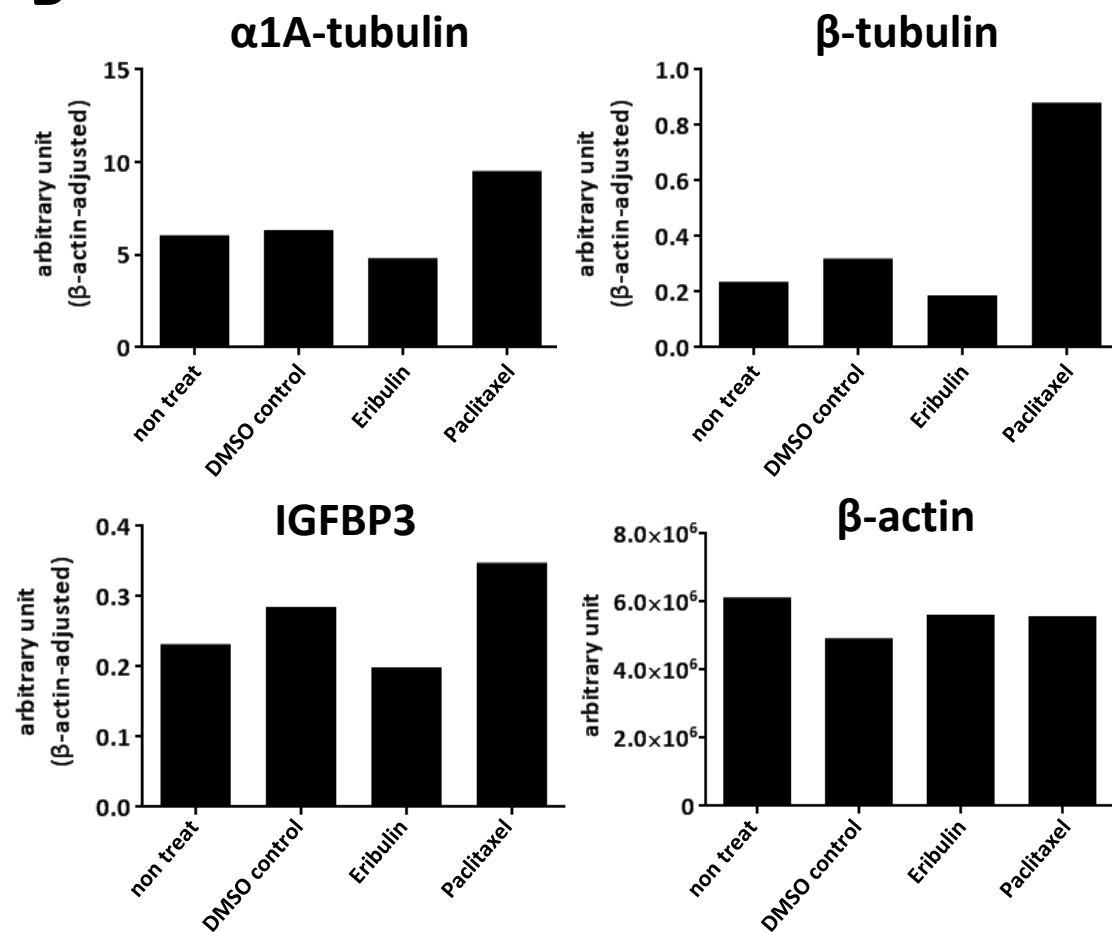

Supplement: Additional file 4 — Western blot analysis of selected proteins in HBVPs treated with eribulin or paclitaxel. HBVPs were treated with eribulin or paclitaxel for 24 hours at 10 × IC50 concentrations as determined in cell proliferation assay. 0.1% DMSO was used as vehicle control. A. Expression levels of several proteins were analyzed by western blot analysis using the antibodies against α1A-tubulin (clone DM1A, Millipore, Billerica, MA), β-tubulin (Santa Cruz Biotechnology, Santa Cruz, CA), IGFBP3 (Santa Cruz Biotechnology) and β-actin (clone AC-15, Sigma Aldrich St. Louis, MO). B. The signals of protein bands were quantified using Multi Gauge version 3.0 software (Fuji Film, Tokyo, Japan). The quantitative data of α1A-tubulin, β-tubulin and insulin-like growth factor binding protein 3 were adjusted by the intensity of β-actin. The calculated values and the intensity of β-actin are also shown. [file 2045-824X-6-3-S4.pdf]

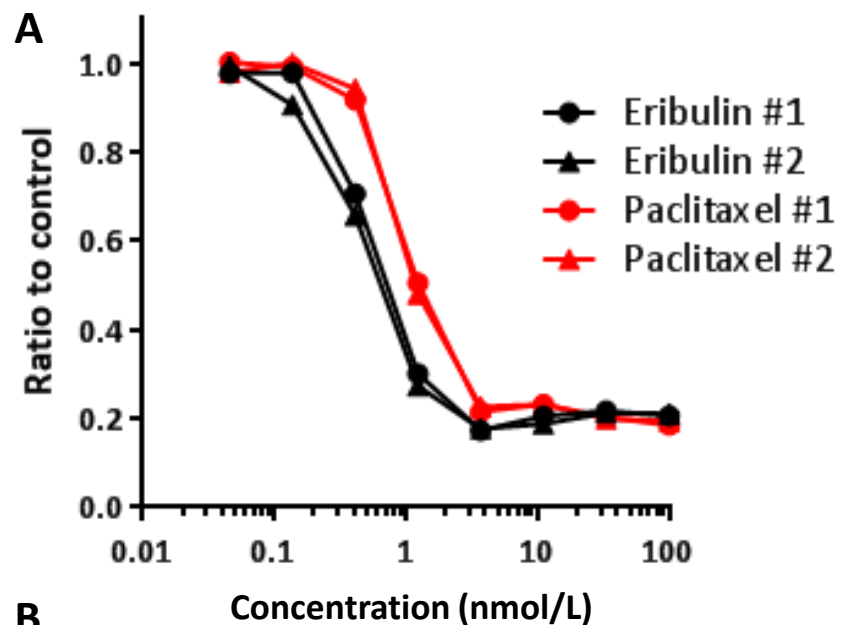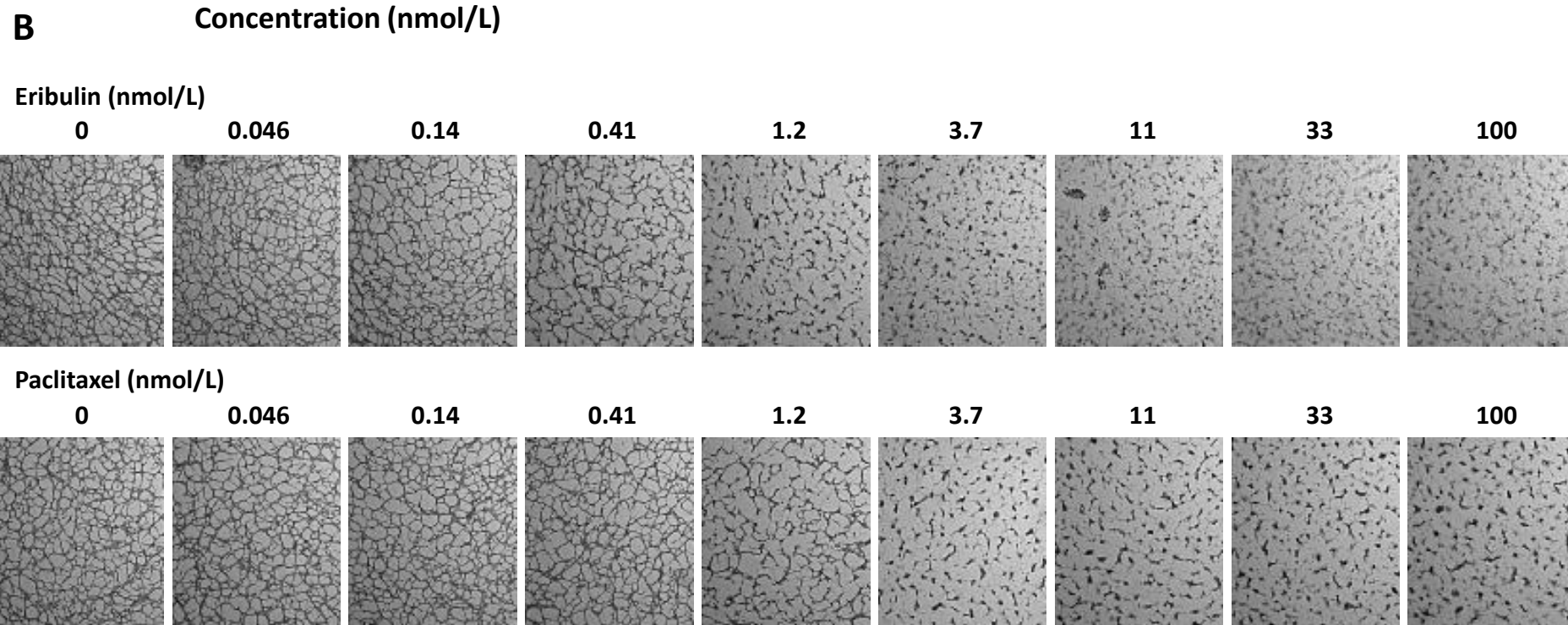

Supplement: Additional file 5 — Effect of eribulin and paclitaxel on angiogenesis in the sandwich tube formation assay. An aliquot (0.4 mL) of the collagen gel mixture (Nitta Gelatin, Osaka, Japan) was added to each well of 24-well plates and allowed to gel at 37°C. HUVECs were harvested by trypsinization, counted and plated onto the gel at 1.5 × 10^5^ cells per well with human endothelial serum free medium (Life Technologies) containing 10 ng/mL of EGF and 20 ng/mL VEGF (assay medium). After overnight incubation at 37°C in a humidified atmosphere containing 5% CO2, medium was removed and 0.4 mL of collagen gel was added to the cells and incubated for 4 hr at 37°C. An aliquot (1.5 mL) of assay medium, containing 0.1% DMSO (as vehicle) or test compounds were added to each well. HUVECs sandwiched in collagen gel were incubated at 37°C in a humidified atmosphere containing 5% CO2 for 4 days and photomicrographs of capillaries were taken with a light microscope using BZ-9000 (Keyence, Osaka, Japan) after adding 0.4 mL of MTT solution. A. Tube length of each capillary was measured using Angiogenesis Image Analyzer software version 2.0 (Kurabo). Assays were performed in duplicate. Both compounds showed close IC50 values: 0.65 - 0.72 for eribulin and 1.18 - 1.26 for paclitaxel. B. Representative images are shown. [file 2045-824X-6-3-S5.pdf]

**A**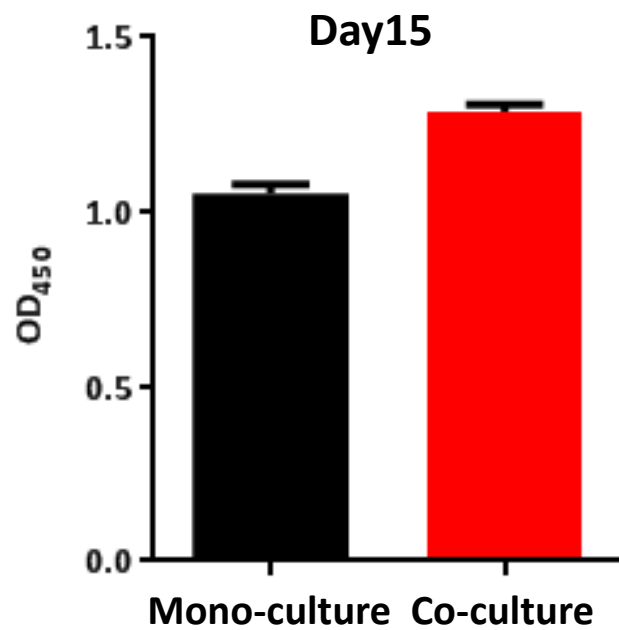**B**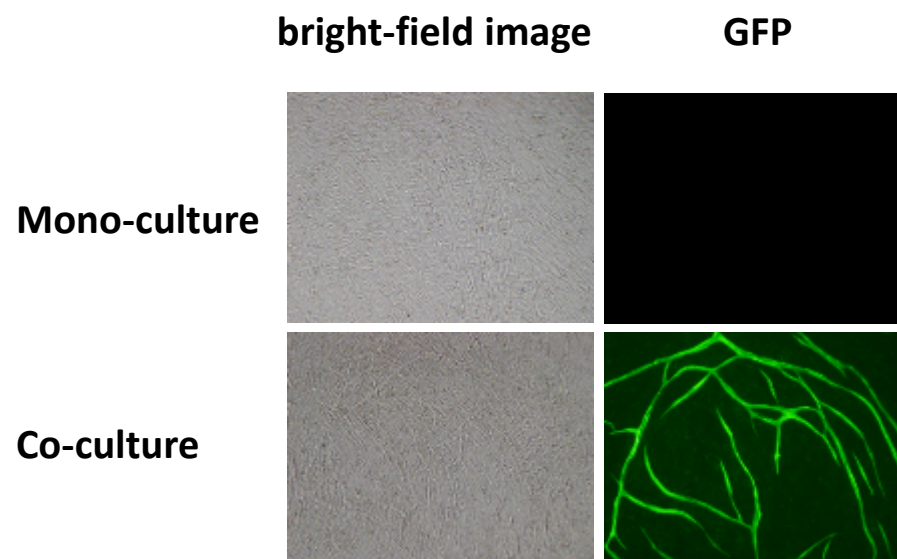

Supplement: Additional file 6 — OD450 values (cell viability) of HBVP mono-culture and HUVEC/HBVP co-culture. In the HBVP mono-culture, HBVPs were diluted to densities of 1.87 × 10^5^ cells/mL. In the HUVEC/HBVP co-culture, HBVPs and AcGFP-expressing HUVECs were diluted and mixed to densities of 1.87 × 10^5^ cells/mL and 1.3 × 10^4^ cells/mL with medium, respectively. Cell suspensions were dispensed at 100 μL per well in 96-well plates and incubated for 15 days with culture medium changes every 2 days. A. Cell viabilities were measured by the WST-8 assay. HUVEC/HBVP co-culture showed slightly higher values compared to HBVP mono-culture, because of HUVEC viability. Data represent means + SEM from three independent experiments. B. Representative images are shown. [file 2045-824X-6-3-S6.pdf]
